# The Role of Recombinant Fibroblast Growth Factor 1 in Enhancing the Angiogenesis in Random Cutaneous Flaps in Animal Model of Rat

**DOI:** 10.29252/wjps.10.2.76

**Published:** 2021-05

**Authors:** Hossein Akbari, Mehdi Ahmadi, Mohammad Javad Fatemi, Ali Foroutan, Peyman Akbari, Hossein Bagheri, Majid Golkar

**Affiliations:** 1 Department of Plastic and Reconstructive Surgery, School of Medicine, Iran University of Medical Sciences (IUMS), Tehran, Iran; 2 Department of Anesthesiology, School of Medicine, Iran University of Medical Sciences, Tehran, Iran.; 3 Department of Parasitology, Pasteur Institute of Iran, Tehran, Iran

**Keywords:** Fibroblast growth factor 1, Angiogenesis, Random skin flap

## Abstract

**BACKGROUND:**

Randomized skin flaps have been used as a basic treatment modality for covering skin defects for a long time but they have always been in the risk of an inherent ischemia. Fibroblast growth factor 1 is a known angiogenic factor in in vitro studies which has shown conflicting results in in vivo investigation. We aimed to determine the effect of recombinant fibroblast growth factor on the angiogenesis rate of random cutaneous flap in animal model of rats.

**METHODS:**

This experimental study was conducted on 24 adult male rats randomized to 2 groups. In the first group FGF1 was injected subdermally in equally divided doses and distances of random flap surface in days 1, 3 and 5. In second group, normal saline was injected as control. Flap surgery was done on day 21 after first injection. The extent of necrosis and angiogenesis (mean vessel density) were assessed in day 14 after surgery.

**Results:**

The mean percentage of clinically apparent necrosis was 35.2% (±10.5) in intervention (FGF1) group and 38.1% (±8.7) in control (normal saline), respectively. Mean vessel density was 86.20±5.6/mm2 in control group and 90.17±5.5/mm2 in intervention group, which showed no statistically significant difference.

**CONCLUSION:**

Mean vessel density and mean percentage of clinically apparent necrosis area were similar in 2 groups of rats with random cutaneous flaps receiving FGF1 or normal saline.

## INTRODUCTION

Inefficient perfusion and subsequent ischemia and necrosis in skin flaps is a leading cause of delayed/impaired healing, flap failure and disappointing results in plastic surgery^1^^.^ Formation of new blood vessels form the endothelium of an existing vascular bed (angiogenesis) in flap starts from 24 hours after surgery and leads to more perfusion, less ischemia and better healing. 

Decreased oxygen concentration in ischemic tissues activate the “angiogenic cascade” by releasing different chemicals including growth factors which bind to vascular endothelial cell surface receptors and increase their mitosis, proliferation, migration and transformation which results ultimately in lumen and basement membrane formation and capillary network expansion^2^^-^^3^.

Random cutaneous flaps are simple, flexible, easy-to-use flaps and are frequently used in reconstructive surgery but these flaps are at an inherent risk of ischemia and necrosis because they have no specific blood vessel and are supplied only by the general vascular network ^4^.

Using exogenous agents for stimulating the angiogenesis in tissues at risk of ischemia and decreasing the rate of necrosis, known as “therapeutic angiogenesis”, has been evaluated in different studies on surgical incisions, burn wounds, cardiac wounds, lacerations, diabetic foots and reconstructive wounds in recent decades. Some studies have shown that accelerating the angiogenesis in cutaneous flaps may also be beneficial but the results are equivocal ^5^^-^^8^.

Fibroblast growth factors (FGFs) first recognized in 1974 refer to a family of large molecules with 22 members, expressed in different tissues from embryonic development to adulthood^9^^-^^11^ (9-11). Acidic FGF (FGF1), initially reported as endothelial cell growth factor (ECGF), was identified several years after the discovery of basic FGF(FGF2) considered as a potent mitogen for vascular endothelial cells and angiogenesis in vitro^12^ (12). In vivo experiences with this angiogen (FGF1) are elusive and its precise practical role in the process of angiogenesis especially in random cutaneous flaps id still unknown^13^ (13). 

This experimental study was designed to assess the role recombinant FGF1 in angiogenesis enhancement in large random skin flaps in rats.

## METHODS

***Ethics statement***

This experimental study was conducted in the Standard Animal Lab of Hazrat Fatemeh Hospital, Tehran, Iran in 2017. The study was approved by institutional Ethics Committee of animal research. It was performed based on our National Institute of Health And National Research Council guidelines for the care and use of laboratory animals.

***Animals and interventions***

Twenty four adult male Wistar rats weighed between 300 and 350 gr were selected and housed individually in their standard experimental cages with free access to food and water, light–dark cycles of 12 h of light and 12 h of darkness and temperature of 22-24 °C. 

Rats were randomly divided to 2 groups. In both groups, the borders of a 1×6 rectangle shape random flap was drawn on the back of the rats. In intervention group, 4 microgram per kg recombinant FGF1 was injected subdermally with a 30-gauge needle and in equally divided spaces in the area of predesigned flap, in days of 1, 3 and 5. In control group, similar amount of normal saline was injected in similar manner as the placebo. After 21 days from first injection, flap surgery was done according to standard surgical and anesthesiologic protocols.

General anesthesia was performed by using 100 mg/kg of intramuscular Ketamine (Alfasan Inc., Woerden, Netherland) and 10 mg/kg intramuscular xylazine (Alfasan Inc., Woerden, Netherland) injected simultaneously. After induction of general anesthesia, the animals were placed in prone position, their limbs were secured by adhesive tape, and their eyes were protected by administration of ophthalmic tears drops. Hair of area was shaved; skin was prepped by alcohol and betadine solution. A standard random flap consisting of epidermis, subcutaneous tissue and panniculus carnosus was raised by blunt dissection and then re-approximated in its original place with 4-0 non-absorbable sutures.

***Hematoxylin eosin (HE) staining***

On postoperative day 14, 24 specimens (0.5×0.5 cm) from the central part of each random flap were obtained and fixed in 4% paraformaldehyde for 24 hours. After fixation, they were transversely cut and embedded in paraffin wax. Four μm of thickness transverse sections were cut from tissue blocks and placed on standard slides for HE staining. 

***Immunohistochemistry (IHC)***

Mean vessel density (MVD) was measured by calculating the number of micro-vessels per unit area (/mm^2^) in immunohistochemically stained specimens. Sections were dewaxed in xylene, rehydrated in ethanol, blocked with H_2_O_2_, rested in sodium citrate buffer, re-blocked with bovine serum albumin and phosphate buffered saline, incubated with primary antibodies, stained, counterstained and photographed. Calculation was made by a light microscope with ×200 magnification (Olympus Corporation, Tokyo, Japan). 7 sections of the central part of flap in each group were prepared, 6 random sites were evaluated in 3 randomly selected sections. Each section was photographed 8 times with a Nikon color digital camera riding on a Nikon Eclipse 80i microscope (Nikon Co., Tokyo, Japan), saved as jpg format. In each image, brown staining was indicative of angiogenesis, counterstain was blue haematoxylin. An average result of stained neovascularized area (MVD) was obtained by averaging the results of 6 to 8 images of each section. 

***Outcome measures***

After 14 days of surgery, gross distal necrosis and capillary count in microscopic evaluation after HE staining were assessed as outcome measures. High-quality digital photographs were taken and flap viability/necrosis was assessed by grid counting. The amount of angiogenesis in flap was estimated by a single pathologist who was blinded to the experimental data. Necrotic tissue starts to becomes demarcated 7 days after surgery and in postoperative day 14, the margins of necrotic area (hard, dark, no bleeding when cut) are very well differentiated from viable areas (soft, white/pink, bleeding when cut). Percentage of necrotic area was calculated by this formula: “necrotic area/total area of flap ×100%”.

***Data analysis***

All statistical analyses were done by SPSS software version 18.0 (IBM Corporation, Armonk, NY, USA). The data of the two groups were compared using an independent-sample t*-*test. P*<*0.05 was considered statistically significant. 

## RESULTS

In day 14 after flap creation day, gross necrotic areas (black and stiff tissue) were well-demarcated and clinically distinguishable. Some degrees of necrosis were seen at distal ends of the flaps in all studied animals. Although there is always a zone of ischemic but viable tissue around the necrotic zone but we evaluated only the apparent necrotic area which could be measured visually without using the microscope.

Gross distal necrosis- Total flap area was 6 cm² in our cases. The mean percentage of clinically apparent necrosis was 35.2(±10.5)% in intervention (FGF1) group and 38.1% (±8.7) in control (normal saline), respectively. Our study showed that mean flap survival area was 4.51(±0.95) cm² in intervention (FGF1) group and 4.32(±0.2) cm² in control (normal saline) group. The difference between flap survival areas was not statistically significant (*P* value=0.17) in 2 studied groups. 

Mean vessel density- Immunohistochemistry studies showed that the mean vessel density was 86.20±5.6/mm^2^ in control group and 90.17±5.5/mm^2 ^in intervention group, which showed no statistically significant difference (*P* value=0.08).

## DISSCUSSION

Occurrence of ischemia and necrosis is a common complication and main cause of flap loss in random cutaneous flap surgery. Different angiogenic factors have been studied to see if they can accelerate the new blood vessels formation and decrease the necrosis and flap failure ratio. FGFs are one of the earliest recognized angiogens which have shown good results in vitro^14^^-^^15^.

Our study evaluated the effect of recombinant FGF1 on angiogenesis in random cutaneous flaps in rat models and showed that FGF1 could not increase the mean vessel density in random cutaneous flaps in rat in comparison with normal saline as control and as a result the mean percentage of clinically apparent necrosis was similar in both groups. Although in our study the overall viability of flaps in FGF1 group was more than control group, but this difference was not statistically significant. This finding may show that angiogenesis and blood supply is increased in FGF1 group but not in a statistically significant manner. Our findings about the FGF1 and random cutaneous flaps are similar to the findings of Fayazzadeh et al who evaluated 2×8 cm dorsal random-pattern skin flaps in 30 rats assigned to 3 groups, after 4 daily subdermal injections of normal saline (control group), FGF1 and FGF2 at designated flap area and showed that mean vessel density and the area of viable flap is statistically significant higher in groups receiving FGF2 than the normal saline group (10 days after flap surgery). They also showed that FGF2 was more potent than FGF in inducing neovascularization in random flaps^16^. 

**Figure 1:**
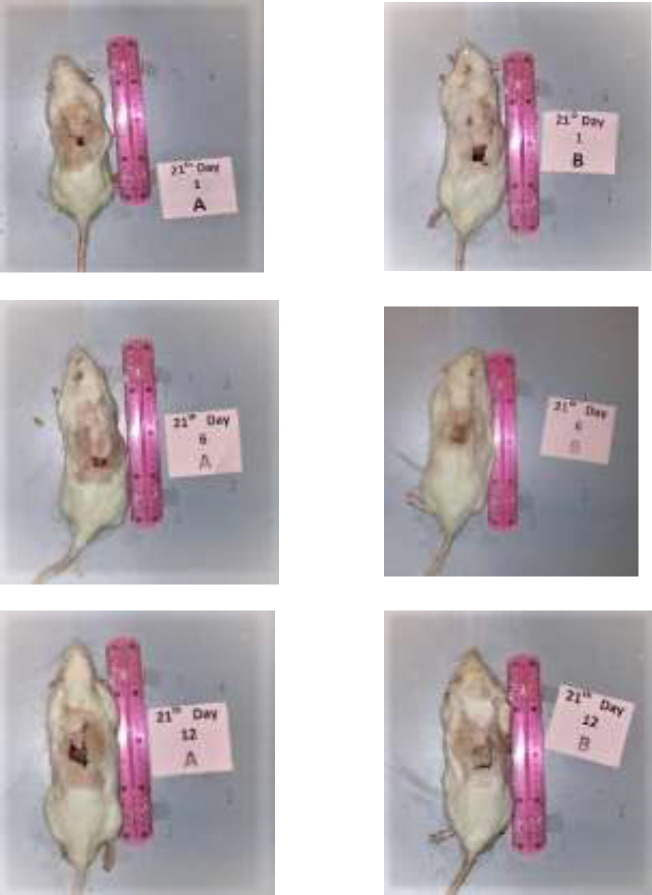
No statistically significant difference was seen in clinically apparent gross distal necrosis between FGF1 (A) and normal saline (B) groups (*P* value=0.17).

But there are other studies with different results. For example, Spyridon et al assessed the 1.5 × 7.5 cm dorsal skin flaps in 45 rats randomized to five groups (receiving injections of saline into fascia of flap, FGF1 subdermally, FGF1 in fascia of recipient bed, FGF1 subdermally in the distal third of the flap, FGF1 into the fascia of the flap). Mean flap survival percentage was 35.4%, 33.7%, 56.3%, 80.4% and 28.3% in these 5 groups respectively and immunohistochemistry analysis showed increased angiogenesis only in groups in which FGF1 was injected in the fascia of recipient bed or subdermally in the distal third of the flap^17^. 

In another study on the 1×4 random flaps on the back of 30 rats randomized into 3 groups of control, erythropoietin group and FGF2 group (all injected subcutaneously in 3 consecutive days), the surface of necrotic area was statistically significant larger in control group than the erythropoietin group and FGF2 group. FGF-2 was more effective than the erythropoietin in accelerating the flap angiogenesis^18^.

Irradiated skin wounds in pigs may have a poor healing due to decreased local level of FGF as increasing the FGF level by intravenous injection of FGF supplements could improve random skin flap viability in these cases^19^. Applying the FGF2 to surface of ischemic flaps in 42 rats showed acceleration in development of connections between the vascular network of tissue bed and cutaneous flap vessels, decreasing the necrosis in highrisk areas and improving the overall survival of the random portion of skin flap^20^.

## LIMITATIONS

Inappropriate dosage, injection intervals, size and shape of flap and technical errors in injecting the FGF (for example injecting in inappropriate depth and sites) may some reasons of our intervention failure in this experiment. We injected 4 microgram/kg FGF1 subdermally and in a distributed manner in our study and didn’t find positive effects on angiogenesis this is while Spyridon et al have shown in their study that when FGF is injected into the fascia of the recipient bed or the subdermal area of the distal third of flap, it is more efficient than when it is injected subdermally and/or in equally divided distances in flap surface area. 

## CONCLUSION

Mean vessel density and mean percentage of clinically apparent necrosis area were similar in 2 groups of rats with random cutaneous flaps receiving FGF1 or normal saline. 
